# Evolutionary relationships of West Nile virus detected in mosquitoes from a migratory bird zone of Colombian Caribbean

**DOI:** 10.1186/s12985-015-0310-8

**Published:** 2015-05-20

**Authors:** Richard Hoyos López, Sandra Uribe Soto, Juan Carlos Gallego-Gómez

**Affiliations:** Molecular and Translational Medicine Group, Medical Research Institute, Faculty of Medicina, Universidad de Antioquia, Medellín, Colombia; Molecular Systematics Research Group, Biosciences School – Sciences Faculty, Universidad Nacional de Colombia, Medellín, Colombia

## Abstract

**Background:**

West Nile virus (WNV) is a member of the genus *Flavivirus*, and it is transmitted between *Culex* sp. mosquitoes and avian hosts. Equids and humans are commonly infected with WNV as dead-end hosts, and the signs and symptoms of infection range from mild illness to neurologic symptoms as encephalitis, meningitis and sometimes death. Previous phylogenetic studies have classified WNV into six genetically distinct lineages and provided valuable insight on WNV dispersal patterns within the Americas and its emergence in different geographic areas. In this study, we isolated, sequenced and genetically characterized the NS5 and envelope genes for two WNV strains detected from Northern of Colombia. Herein we describe the evolutionary relationships with representative WNV-strains isolated in a variety of epidemic outbreaks and countries, to define the phylogeographic origin and possible implications in the epidemiology of this emergent virus in Colombia.

**Findings:**

Fragments of the NS5 and Envelope genes were amplified with RT-PCR and sequenced to obtain 1186-nt and 1504-nt portions, respectively. Our sequences were aligned with 46 sequences from WNV-strains collected in the U.S., Mexico and Argentina for phylogenetic reconstruction using Bayesian methods. Sequence analyses identified unique non-synonymous substitutions in the envelope gene of the WNV strains we detected, and our sequences clustered together with those from the attenuated Texas – 2002 genotype.

**Conclusions:**

A new strain closely related to attenuated strains collected in Texas during 2002 was identified from Colombia by phylogenetic analysis. This finding may explain the absence of human/equine cases of WNV-encephalitis or severe disease in Colombia and possibly other regions of South America. Follow-up studies are needed in ecosystems used by migratory birds areas and virological/entomological surveillance.

## Findings

### Introduction

West Nile virus (WNV) is a member of the Japanese encephalitis antigenic complex (JEV) within the *Flavivirus* genus and is transmitted by *Culex* spp. mosquitoes among birds. Other vertebrates such as mammals and reptiles also become infected [1]. Human infection with WNV causes mild to severe illness, sometimes affecting the nervous systems and provoking encephalitis, meningitis and death [2].

Since the first reports of WNV isolation from Africa, Europe, India, Russia, Israel, France, and its 1999 introduction into North America, WNV has extended its geographic distribution throughout the United States [3]. Subsequently, in the following years this arbovirus was detected in Canada, Mexico, Guatemala, Caribbean islands, and South America [4–6]. Serologic evidence for the natural circulation of WNV in Colombia has been observed in equids sampled from the department of Córdoba [7, 8] and other regions from the Caribbean [9–11]. Despite WNV being isolated for the first time from captive flamingoes in Santa Fé Zoo (Medellín, Colombia) [12], it is not clear why WNV has not been isolated from or been the cause of detectable disease in horses or humans in Colombia. Possible explanations include: circulation of WNV in remote enzootic cycles away from human settlements, limited vector competence of mosquito species, ornitophilic blood-feeding preferences, cross-protective immunity in humans from other flaviviruses (dengue, Saint Louis encephalitis viruses), or the circulation of WNV-strains with low or attenuated virulence [10–12].

WNV occurs in four major lineages, but lineage 1 is epidemiologically relevant. Lineage 1 is subdivided into three clades (1a, 1b, 1c); clade 1a contains isolates from Africa, Europe, the Middle East, Russia and Americas [1, 3]. WNV-American strains have close relationships with three Old World isolates: IS98-ST1 (Israel - 1998), PaH001 (Tunez-1997) and goose-03 (Hungary-2003), and extensive studies have allowed detailed investigations of WNV microevolution in different areas over time and also the emergence of new genotypes [3]. In this sense, phylogenetic analysis has enabled the understanding of epidemiological patterns of emergence, dispersal routes, adaptation to new hosts/mosquitoes species, and spatio-temporal patterns of evolution [1, 3, 5, 12]. Evolutionary studies are necessary to identify “drivers” of emergence, molecular evolution of virulence and associations to ecological factors that allow the establishment of this arbovirus pathogen in human populations.

Between 2011 and 2013, a surveillance study was performed at one locality that is characteristic of a large migratory bird population in northern Colombia. We detected WNV in pools of mosquitoes and amplified two viral regions that were used to establish the phylogenetic relationships with strains isolated in the U.S., Mexico and Argentina, with the goal of defining the evolutionary relationships with genotypes previously isolated from outbreaks in the geographic areas mentioned above.

## Material and methods

### Samples

**Fig. 1:**
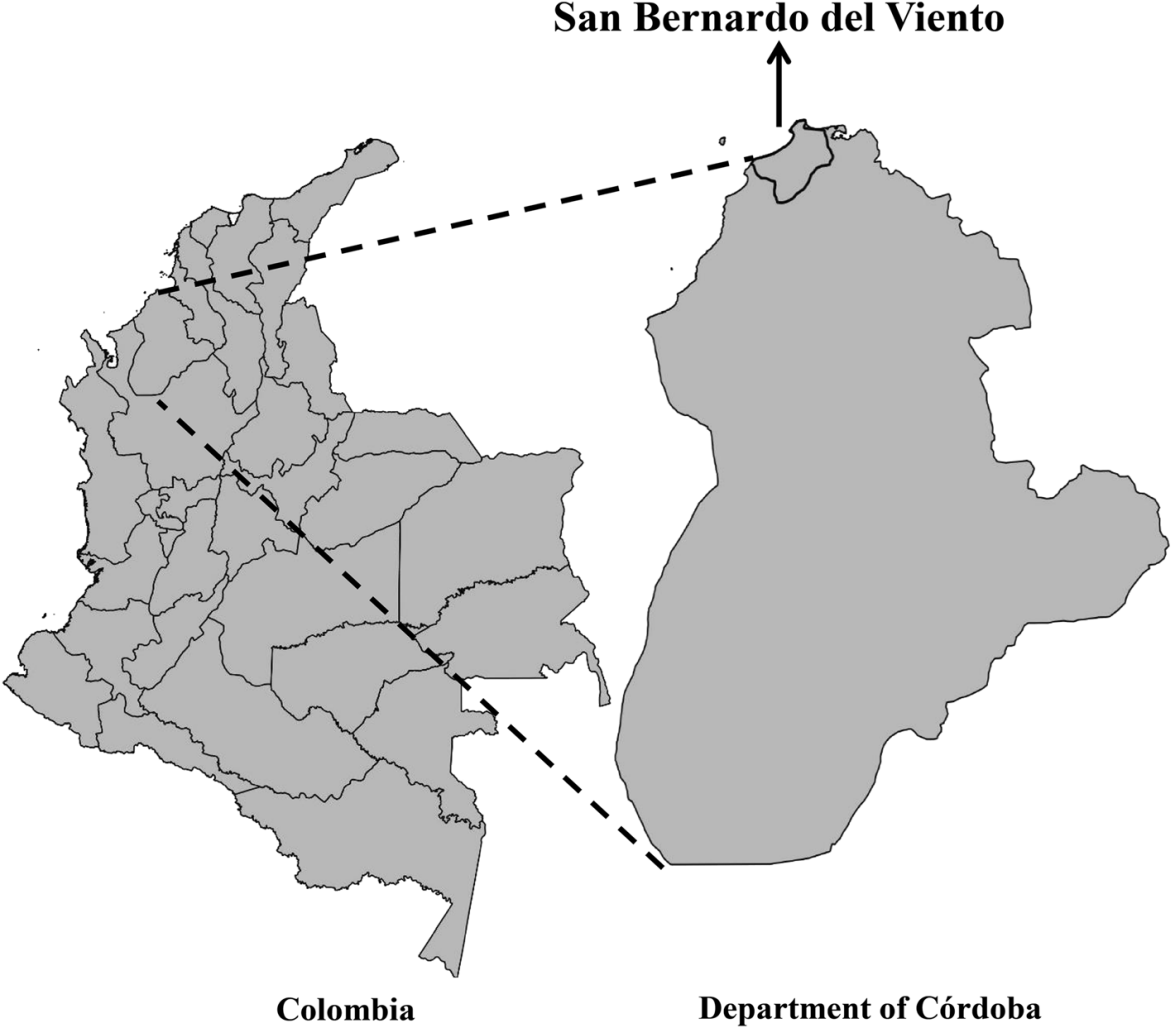
Map of the study area showing San Bernardo del Viento (Córdoba, Colombia)

During virological surveillance for detection of emerging and re-emerging arboviruses between 2011–2013 in San Bernardo del Viento (Córdoba, Colombia) (9° 21´ 30.97” N, 75° 58´ 37.28” W) (Fig. 1), mosquitoes were collected using CDC-light/EVS traps that were baited with dry ice (CO_2_). All the insects sampled were separated into pools through morphological identification and triturated using minimum essential medium (MEM) supplemented with 10 % fetal bovine serum, 1 % penicillin, then clarified by centrifugation at 13,000 rpm for 30 min. Supernatant was used for RNA extraction and generic nested reverse transcriptase polymerase chain reaction (RT-PCR) for detection of flaviviruses (Table 1) [13]. Two mosquito pools, each containing 40 and 50 specimens (whose mosquitoes had a high percentage −99.57 % of similarity in their DNA Barcode sequences with *Culex* (*Melanoconion*) *erraticus*) were positive for a member of the *Flavivirus* genus. Sequencing of PCR-products, BLASTN and Neighbor-Joining phylogenetic analysis allowed identification of these sequences as West Nile virus.

**Table 1:** Primers and RT-PCR (generic/nested) conditions for arboviral detection in target groups

Arboviruses group	Primers	Conditions for RT-PCR	Reference
Flavivirus	Flavi1+ GAYYTIGGITGYGGIIGIGGIRGITGG	1 cycle – 45 min/38°C	[13]
	Flavi1− TCCCAICCIGCIRTRTCRTCIGC	40 cycles: 30 sec/94°C, 1 min/47°C, 75 sec/68°C	
		1 cycle – 5 min/68°C	
	Flavi2+ YGYRTIYAYAWCAYSATGGG	1 cycle – 2 min/94°C	
	Flavi2− CCARTGITCYKYRTTIAIRAAICC	40 cycles: 30 sec/94°C, 1 min/47°C, 15 sec/72°C	
		1 cycle – 5 min/72°C	

### Molecular protocols

Total RNA was extracted from the PCR-positive homogenates with the RNeasy kit (Qiagen, Valencia) and used for RT-PCR using the One-Step RT-PCR Kit (Qiagen, Valencia, CA) as described previously [14, 15]. Two viral genes were amplified: NS5 (primers: FU1PMF-TACAACATGATGGGVAARAGWGARAA/cFD3PMR-ARCATGTCTTCYGTBGTCATCCA) and Envelope (primers: WN1101-GATGAATATGGAGGCGGTCA/WN1816A-CCGACGTCAACTTGACAGTG and WN1751-TGCATCAAGCTTTGGCTGGA/WN2504A TCTTGCCGGCTGATGTCTAT).

### Sequence analysis

A total of 46 genome sequences of envelope/NS5 from WNV isolates, representative of American clade 1a-Cluster 4 geographic locations [16], were downloaded from GenBank (Table 2). Nucleotide sequences were aligned using the MAFFTv7.0 (http://mafft.cbrc.jp/alignment/server/) and then transferred in FASTA format to BioEdit (http://www.mbio.ncsu.edu/BioEdit/bioedit.html) for manual editing, keeping gaps consistent within the reading frame. The sequences of NS5 and envelope of the WNV-strains were aligned and evaluated in jModelTest v2.1.4 [17] using the Akaike criterion informative for identify appropriate substitution model nucleotide. One test was also performed for concatenated sequences. The XML file for Bayesian analysis was created in BEAUti v1.5.4 (http://www.molecularevolution.org/software/phylogenetics/beauti), describing model of sequences, invariants, gamma distribution, size of the chain run (20 million of generations), coalescent constant population and for accommodate the variation in substitution rate among branches, a random local clock model was chosen for this analysis [18].

**Table 2:** Sequences downloaded of Genbank and background information of WNV strain/isolates used in this study

Accession number	Strain	Location	Host-species	Year
GQ379160	ArEq001	Argentina	Horse	2006
GQ379161	ArEq003	Argentina	Horse	2006
DQ118127	goose-Hungary/03	Hungary	goose	2003
AF481864	IS-98 ST1	Israel	sick stork	1998
DQ080065	TVP9221	Mexico:Baja Calfornia Norte	Grackel	2003
DQ080064	TVP9222	Mexico:Baja Calfornia Norte	Coot	2003
DQ080063	TVP9223	Mexico:Baja Calfornia Norte	Pigeon	2003
DQ080066	TVP9220	Mexico:Baja Calfornia Norte	Cormorant	2003
DQ080068	TVP9218	Mexico:Baja Calfornia Norte	Blue Heron	2003
DQ080067	TVP9219	Mexico:Baja Calfornia Norte	Green Heron	2003
DQ080070	TVP9115	Mexico:Sonora	Grackel	2003
DQ080069	TVP9117	Mexico:Tamaulipas	Horse	2003
DQ164201	AZ 2004 (Arizona 2004)	USA: Arizona	Human- plasma	2004
DQ080057	CA-03 Arcadia-S0331532 (I)	USA: California, Los Angeles	Crow	2003
DQ080058	CA-03 Arcadia-S0334814 (J)	USA: California, Los Angeles	Crow	2003
DQ080072	FL232	USA: Florida, Palm Beach Co.	Catbird	2001
DQ080071	FL234	USA: Florida, Sumter Co.	Horse	2002
GU827998	Bird114	USA: Harris County, Texas	blue jay	2002
GU828002	v4095	USA: Harris County, Texas	*Culex quinquefasciatus*	2003
GU828000	Bird1175	USA: Harris County, Texas	Blue jay	2003
GU828003	Bird1881	USA: Jefferson County, Texas	Mourning dove	2003
AF404753	MD 2000-crow265	USA: Maryland	Crow	2000
AY795965	ARC10-02	USA: Michigan	Human- plasma	2002
GU828004	Bird1519	USA: Montgomery County, Texas	Bluejay	2003
DQ211652, AY842931	NY99 385-99	USA: New York	Snowy Owl	1999
AF196835	NY99-flamingo382-99	USA: New York	Flamingo	1999
AF202541	HNY1999	USA: New York	Human	1999
AF260967	NY99-eqhs	USA: New York	Horse	1999
DQ164189	NY 2003 Albany	USA: NY, Albany	American crow	2003
DQ666452	BSL2-05	USA: South Dakota	Human- plasma	2005
DQ164198	TX 2002 1 (80025)	USA: Texas	Human- plasma	2002
DQ164205	TX 2002 2 (80022)	USA: Texas	Human- plasma	2002
AY712945	Bird 1153 (TWN274)	USA: Texas	Mourning dove	2003
AY712946	Bird 1171 (TWN269)	USA: Texas	Blue jay	2003
AY712948	V4369 (TWN382)	USA: Texas	*Culex quinquefasciatus*	2003
DQ080053	AZ-03 03-1799	USA:Arizona, Apache Co.	*Culex tarsalis*	2003
DQ080051	AZ-03-1623 (A)	USA:Arizona, Cochise Co.	*Culex tarsalis*	2003
DQ080052	AZ-03-1681 (B)	USA:Arizona, Maricopoa Co.	*Culex tarsalis*	2003
DQ080055	CA-03 IMPR 102 (F)	USA:California, Imperial Valley	*Culex tarsalis*	2003
DQ080056	CA-03 IMPR-1075 (G)	USA:California, Imperial Valley	*Culex tarsalis*	2003
DQ080054	CA-03 GRLA-1260	USA:California, Los Angeles	*Culex quinquefasciatus*	2003
FJ527738	LSU-AR01	USA:Louisiana	Blue jay	2001
DQ080062	LA02-2829 (TWN165)	USA:Louisiana	Mosquito	2002
DQ080061	Bird2409 (TWN 496)	USA:Louisiana	Cardinal	2004
HQ671697	BID V4197-2001	USA: Connecticut	*Aedes vexans*	2001
KJ501528	BID V6697-2002	USA	Blue jay	2002
AY646354	USA	USA: New York	Human-plasma	2002
DQ164199	TX-2003	USA: Texas	Human	2003
AY660002	Mex03 (TM171-03)	Mexico: Tabasco	Raven	2002
JN716371	COL524-08	Colombia: Antioquia, Medellín	Flamingo	2008
JN716372	COL9835-08	Colombia: Antioquia, Medellín	Flamingo	2008

Bayesian phylogenetic analysis was performed using the BEAST software package v2.1.3 [19], and estimation of the maximum clade credibility (MCC) phylogenetic tree was achieved using TreeAnnotator-v2.0.2. BEAST output was viewed with TRACERv-1.5 and evolutionary trees were generated in the FigTree-v1.3.1.

DNAsp-v5.0 [20] was used to establish polymorphic sites between NS5/envelope sequences characterized in our study and reference sequences of representative WNV strains.

## Results and discussion

The two pools infected with WNV, corresponding probably to the mosquito species *Culex* (*Melanoconion*) *erraticus* (codes: CDCCA4-12 – CDCCZ2-21), were collected in September, 2012 from coastal mangroves in a migratory bird zone located in San Bernardo del Viento (Córdoba Department). Both isolates were RT-PCR amplified, including one sequence of 1504 nt in length from the envelope gene (Genbank accession number: KM212943 – KM212944) and another 1186 nt long from the NS5 gene (Genbank accession number: KM212941 – KM212942). jModelTest-v2.1.4 estimated the same model of substitution nucleotide, General-Time-Reversible (GTR) + gamma distribution (−lnL = 4792.34, AICc = 9798.9686) for both viral regions. A concatenated file with two NS5/envelope sequences (2690 nt) estimated the same model and was used for phylogenetic inference.

**Fig. 2:**
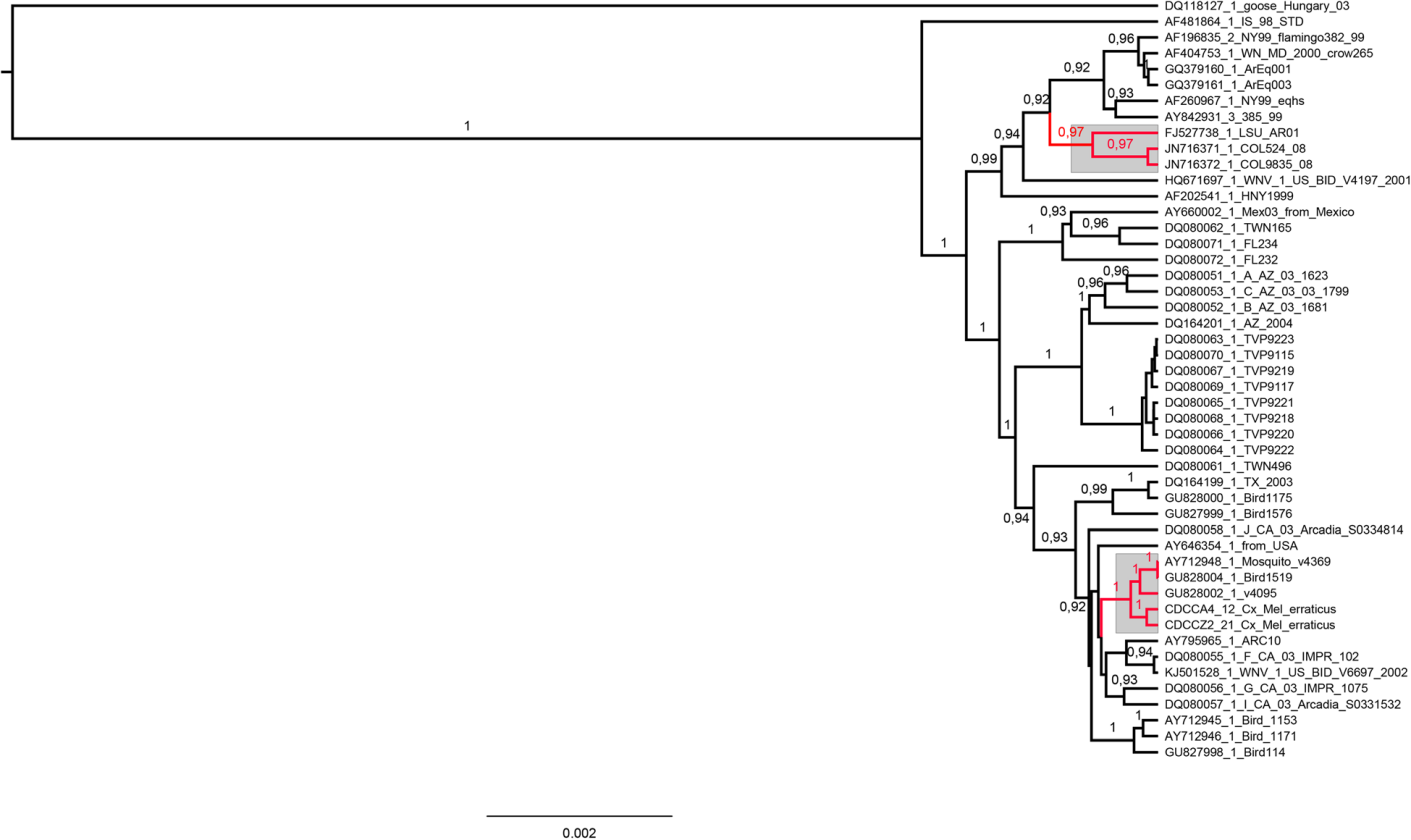
Phylogenetic tree estimated by Bayesian analysis of 46 sequence strains (NS5-Envelope) of West Nile virus data under the GTR + G model of nucleotide substitution. The number accession of Genbank is followed by abbreviated name of WNV-strain. The branches in red belong to characterized previously by Osorio et al. (2012) and characterized by ours

The phylogenetic tree inferred with Bayesian methods indicated that our two WNV-samples were closely related to strains Mosquito-v4369, Bird1519 and v4095, all belonging to southeastern coastal Texas genotype (Fig. 2). Our sequences differed from that of this WNV-genotype in three positions in the envelope gene: only two positions are unique (shaded positions: 547,641) and position 555 shared the same nucleotide with strains HNY1999, COL524 and COL9835. These substitutions were all synonymous. No unique mutations were found in NS5, but our sequences shared similar differences to the WNV-Texas genotype and other WNV genotypes (Table 3). The southeastern coastal Texas genotype includes several isolates collected in 2002 from Texas, and is considered to have an attenuated phenotype with a small plaques (sp) size, temperature sensitivity (ts), reduced replication in cell culture and reduced neuroinvasiveness that is dose-dependent [21]. The southeastern coastal Texas genotype has not been detected since 2002, suggesting its possible extinction [3]. In Colombia, previous work characterizing WNV-strains isolated from flamingoes (COL524/COL9835) showed a close genetic relationship with WNV strains isolated in Louisiana in 2001 and the NY99 strain (Fig. 2), but *in vitro* phenotypic characterization showed differences with the attenuated Texas-genotype. In fact, COL524/COL9835 has high virulence in chicken eggs and newborn/4-week-old Balb/c mice [12].

**Table 3:** Polymorphic sites between envelope and NS5 sequences belonging to southeastern coastal Texas genotype and our samples from Northern of Colombia (Córdoba, San Bernardo del Viento)

Sequences	Polymorphic sites	
	Envelope	NS5
	1	11222222
	22455695	57012222
	834745480	91811266
	440675140	93524727
AF202541.1 HNY1999	TGCTTGAGC	TCCTTCCG
JN716371.1 COL524	CAT....T.	…C..T.
JN716372.1 COL9835	CAT......	…C..T.
AY712948.1 Mosquito-v4369	CATC.A..T	CTT..TT.
GU828004.1 Bird1519	CATC.A..T	CTT..TTA
GU828002.1 v4095	CATC.A..T	C.T.CTT.
CDCCA4-12 Cx.erraticus	CATCC.G.T	C.T..TT.
CDCCZ2-21 Cx.erraticus	CATCC.G.T	C.T..TT.

Number represented polymorphic positions inside alignment of envelope and NS5 sequences and dots indicate sequence homology. Nucleotides in shadow evidenced unique substitutions that distinguish WNV-detected in mosquito pools from other strains

Our results demonstrate genetic diversity of WNV strains circulating in Colombia. The presence of the attenuated coastal Texas genotype could explain, in part, the lack of human and equine cases detected. Previous work has shown WNV-seropositivity in horses from the Caribbean region with no disease reported [7–11]. Different WNV genotypes may converge along migratory bird flyways, which pass from the U.S. to Mexico, Colombia and Venezuela, Caribbean Islands, and other parts of South America south to Argentina. The high diversity of migratory birds in certain areas of Caribbean Colombia [3, 4, 12, 22] may be important for WNV maintenance.

Finally, our results indicate a close evolutionary relationship with the attenuated coastal Texas genotype requires further studies in cell culture and animal models to confirm the attenuated phenotype. Additional surveillance focused on avian and mosquito fauna is also needed to obtain more isolates of WNV in conserved Colombian ecosystems to further examine the genetic diversity of WNV and possible strain dissemination to another geographic areas.
